# Improvement in Redox Homeostasis after Cytoreductive Surgery in Colorectal Adenocarcinoma

**DOI:** 10.1155/2021/8864905

**Published:** 2021-07-31

**Authors:** Salome Sadat Salehi, Hossein Mirmiranpour, Soghra Rabizadeh, Alireza Esteghamati, Giovanni Tomasello, Abbas Alibakhshi, Niloofar Najafi, Armin Rajab, Manouchehr Nakhjavani

**Affiliations:** ^1^Endocrinology and Metabolism Research Center (EMRC), Vali-Asr Hospital, Tehran University of Medical Sciences, Tehran, Iran; ^2^Department of Biomedicine, Neurosciences and Advanced Diagnostics, University of Palermo, Palermo, Italy; ^3^Department of General Surgery, Tehran University of Medical Sciences, Tehran, Iran

## Abstract

Colorectal cancer (CRC) as one the most common cancer type is associated with oxidative stress. Surgery is the only curative modality for early-stage CRC. The aim of this study was to evaluate the oxidative damage biomarkers as well as enzymatic and nonenzymatic antioxidants in patients with CRC before and after tumor resection and in healthy controls. 60 patients with stage I/II colorectal adenocarcinoma and 43 healthy controls were recruited in this study. We measured plasma levels of oxidative damage biomarkers, including advanced oxidation protein products (AOPP), advanced glycation end products (AGEs), malondialdehyde (MDA), and oxidized low-density lipoprotein (ox-LDL) at baseline and after tumor removal. We also evaluated the plasma activity of superoxide dismutase (SOD), catalase (CAT), and glutathione peroxidase (GPx) as enzymatic antioxidants and the ferric reducing antioxidant power (FRAP) assay for nonenzymatic antioxidant capacity. Patients with CRC had significantly higher AGE, AOPP, MDA, and ox-LDL and also FRAP levels and higher SOD and GPx and lower CAT activity levels compared to healthy controls (*p* < 0.05). We did not observe any statistically significant correlation between redox biomarkers and the size and stage of the tumor. AGEs (72.49 ± 4.7 vs. 67.93 ± 8.8, *p* < 0.001), AOPP (137.64 ± 21.9 vs. 119.08 ± 33.1, *p* < 0.001), MDA (3.56 ± 0.30 vs. 3.05 ± 0.33, *p* < 0.001), and ox-LDL (19.78 ± 0.97 vs. 16.94 ± 1.02, *p* < 0.001) concentrations reduced significantly after tumor removal. The largest effect sizes were found in ox-LDL (*d* = −2.853, 95% CI 2.50-3.19) and MDA (*d* = −1.617, 95% CI 0.43-0.57). Serum FRAP levels (1097.5 ± 156.7 vs. 1239.3 ± 290, *p* < 0.001) and CAT (2.34 ± 0.34 vs. 2.63 ± 0.38, *p* < 0.001), GPx (102.37 ± 6.58 vs. 108.03 ± 6.95, *p* < 0.001), and SOD (5.13 ± 0.39 vs. 5.53 ± 0.31, *p* < 0.001) activity levels increased significantly after surgery. The largest effect sizes among antioxidants were seen in SOD (*d* = 1.135, 95% CI 0.46-0.34) and GPx (*d* = 0.836, 95% CI 0.35-0.23). This study indicated that patients with colorectal cancer had higher levels of oxidative stress and antioxidant activity compared to healthy controls. After surgical resection of tumor, we observed a substantial improvement in redox homeostasis.

## 1. Introduction

Colorectal cancer (CRC) is one of the most common malignant cancers and the second leading cause of cancer-related death worldwide [1]. Nutritional oxidative stress and inflammation of the colonic mucosa play an essential role in the molecular mechanism of CRC [2]. High incidence of cancer in colorectal area, compared to other regions of the GI tract, may be explained by intracolonic free radical formation [3]. Considerable research is being done in redox and has revealed that the oxidative stress is closely related to all aspects of cancer, from carcinogenesis to tumor progression and from prevention to treatment. Cancer initiation and progression have been linked to oxidative stress by increasing DNA mutations or inducing DNA damage, genome instability, and cell proliferation [4–6]. Oxidative stress is a cellular phenomenon which occurs as a result of physiological imbalance between the levels of antioxidants and oxidants (free radicals or reactive species) in favor of oxidants [7–9]. Reactive oxygen species (ROS) are produced naturally in normal cell activity while tumor cells continuously generate ROS at increased levels [10]. ROS overproduction in cancer cells can damage cellular structure and function via oxidation of DNA, proteins, and lipids [11, 12]. Antioxidants are the first line of defense against free radical damage and are essential for maintenance of redox homeostasis [13]. However, high level production of ROS in cancer cells could overwhelm antioxidant capacity and functionality and might be involved in neoplastic transformation and disease progression [14]. Cancer cells are located in a very complex microenvironment together with stromal components which is interposed between the malignant cells and normal host tissues [10, 15]. The tumor microenvironment (TME) is composed of malignant and nonmalignant cells, connective tissue, and vasculature. It plays an essential role in tumor initiation, progression, and metastasis [16]. Surgery is the primary strategy for treatment of potentially curable (early and locally advanced) colorectal cancer [17–19]. Adjuvant therapy is most often needed to reduce the risk of relapse and increase survival [18]. Imbalanced redox status created by cancer cells' rapid growth and limited availability of nutrients and oxygen render them insensitive to further stress inducers, such as chemotherapy and radiation, and significantly associated with poorer prognosis and premature mortality [20, 21]. The aim of this study was to evaluate the oxidative damage biomarkers as well as enzymatic and nonenzymatic antioxidants in CRC patients before and after cytoreductive surgery compared to healthy controls. Moreover, since imbalanced redox status is associated with tumor progression, resistance to therapy, and increased risk of recurrence [10, 22], this research has mainly focused on whether the surgical removal of the tumor microenvironment affects systemic oxidative stress in patients with CRC.

## 2. Materials and Methods

### 2.1. Study Groups

This hospital-based study involved 60 patients with early-stage colorectal cancer who were candidates for curative surgery and 43 controls who were healthy volunteers from hospital staff. Patients and controls were matched according to age, gender, and body mass index (BMI). From these patients, five were detected via screening and 55 were diagnosed as having symptoms of CRC.

Sample size was calculated using the following formula [23]:

(1) $$\begin{array}{c} \text{Sample size }\left(N\right)=2\text{S}{\text{D}}^{2}\;\frac{{\left(Z_{\alpha /2}+Z_{\beta }\right)}^{2}}{d^{2}}, \end{array}$$

where SD is the standard deviation based on previous studies or pilot studies, *Z*_*α*/2_ = 1.96 (from the *Z* table) at type I error of 5%, *Z*_*β*_ = 0.842 (from the *Z* table) at 80% power, and *d* is the effect size which is the difference between mean values.

#### 2.1.1. Inclusion Criteria

Cases that met the following criteria were included: patients with pathologically confirmed diagnosis of colorectal adenocarcinoma or mucinous adenocarcinoma, either well-differentiated or moderately differentiated, stage І or II according to the TNM classification [24] as early stages of CRC [25], without local or distant metastasis, with normal carcinoembryonic antigen (CEA) level, with no family history of colorectal cancer, and no history of prior colorectal cancer or operations. All participants had no history of smoking at least 4 weeks prior to the study. Only patients and healthy controls with normal laboratory evaluations including complete blood count (CBC), vitamin D levels, and kidney, liver, and thyroid function tests (hemoglobin ≥ 10 and albumin ≥ 3.5 were required for surgery) were included. None of the patients and healthy controls had any known disease including malignancy, autoimmune disease, infectious disease, cardiovascular disease, anemia, diabetes, or hyperlipidemia.

#### 2.1.2. Exclusion Criteria

Since radiation and chemotherapy can result in the production of free radicals, reduced antioxidant activity, and increased oxidative stress [26], patients were excluded if they had undergone preoperative radiotherapy or neoadjuvant chemotherapy. Patients who received blood during surgery were also excluded. Patients and healthy controls who had a history of drugs associated with drug-induced oxidative stress including nonsteroidal anti-inflammatory drugs (NSAID), antiretroviral agents, antipsychotics (chlorpromazine) [27, 28], and corticosteroids [29] and also drugs with antioxidant effects like dietary supplement for the last 2 weeks were excluded.

### 2.2. Clinical Evaluation

All participants' demographic and anthropometric data including age, gender, height, and weight in light clothing was recorded. Height was measured in standing position by a standard stadiometer and with the approximation of 0.1 cm. Weight was measured by a digital scale (Beurer, GS49, Germany). Blood pressure was measured using a standard sphygmomanometer (Riester, Big Ben adults, Germany) in the sitting position after 10 min rest and was repeated after 5 minutes, and the mean value was recorded. The body mass index (BMI; kg/m^2^) was calculated according to the Quetelet formula.

### 2.3. Pathology Data

Tumor information was collected from presurgery imaging and biopsy pathology, intraoperative inspection and pathology, and postexcision pathology reports. All tumor characteristics including stage, histological type, pathological grading, lymph node involvement, and tumor size and location were recorded accordingly.

### 2.4. Perioperative Care

All patients were admitted the day before the procedure to the surgical ward of the clinical department of general and gastroenterological surgery at Vali-Asr Hospital, affiliated with Tehran University of Medical Sciences. All patients were educated regarding what they should expect in the pre- and postoperative phases of their surgery. Mechanical and antibiotic bowel preparation was started the day before surgical intervention. Laparotomic (open) curative resection surgery was performed through a midline incision by the same surgical team. After surgery, all patients received analgesics and were monitored continuously with blood pressure, pulse rate, and oxygen saturation measurements.

### 2.5. Blood Sampling

After 8 to 10 hours of overnight fasting, 10 mL of blood sample was obtained from controls and all patients 24 hours before and after surgery. In major surgical procedures, increases in surgery-induced oxidative stress occur acutely [30], and as shown in majority of the studies, the redox status of the body is tightly regulated and returns to normal within 24 hours of surgery [31–33]. Furthermore, oxidative stress biomarkers have short half-life (mostly less than 24 hours) in the plasma [7, 31, 34–39]. In the presence of proper incisional pain management [32] and in the absence of colorectal surgery complications, 24 hours postsurgery might be an appropriate time for the earliest evaluation of tumor removal outcome on redox status with less confounding effect of surgical stress. Following blood sampling, the blood was centrifuged at 2500 × g for 10 min, and then, the serum was separated and aliquoted into tubes and were stored at −70°C until assayed.

### 2.6. Redox Biomarker Measurement

We measured plasma levels of oxidative damage biomarkers, including advanced oxidation protein products (AOPP), advanced glycation end products (AGEs), malondialdehyde (MDA), and oxidized low-density lipoprotein (ox-LDL) in healthy controls and in patients at baseline and after tumor removal. The blood samples also were checked for plasma activity of enzymatic antioxidants including superoxide dismutase (SOD), catalase (CAT), and glutathione peroxidase (GPx) and the ferric reducing antioxidant power (FRAP) assay for nonenzymatic antioxidant capacity. Experiment for AGEs, AOPP, and FRAP was performed in triplicate. If the concentration was higher than the dynamic calibration range, the samples were measured with dilution and the values were multiplied by the dilution coefficient. If the concentration was lower, the measurement was repeated. Samples measured by kits were duplicated.

#### 2.6.1. Oxidative Damage Biomarkers

Measurement of ox-LDL level was performed using a commercially available sandwich ELISA method (Mercodia, Uppsala, Sweden) with intra-assay CV%: 3.4, inter-assay CV%: 7.2 (U/L), and detection limit: 0.6 (mU/L), ranging from 1.4 to 22.5 (mU/L). Serum MDA level was measured using a colorimetric method (Cayman, USA) with intra-assay CV%: 5.2 and inter-assay CV%: 5.7, with a range of 1.83 to 3.94 (*μ*mol/L). AOPP was determined according to the spectrophotometric method of Kalousova et al. (FLUOstar OPTIMA, BMG, Germany). In detail, 200 *μ*L of serum was diluted by a factor of 5, in PBS. Additionally, 200 *μ*L of chloramine T (0-100 *μ*mol/L) for calibration and 200 *μ*L of PBS as blank were also added to different microplates. Finally, 10 *μ*L of acetic acid and 20 *μ*L of 1.16 M potassium iodide (KI) were added to preparations. Measurements were made at absorbance of 340 nm and were expressed in *μ*mol/L. The intra-assay coefficient of variation was less than 5%, and the inter-assay coefficient of variation was less than 10%. AGEs were assessed using spectrofluorimetric assay as described by Kalousova et al. [40]. Patients' sera were diluted by a factor of 50 in phosphate-buffered saline (PBS). Fluorescence intensity at 350 nm excitation and 440 nm emission was recorded and was expressed as percentage of fluorescent emission. The intra-assay coefficient of variation was 5.1% and inter-assay was 7.9%.

#### 2.6.2. Antioxidants

Serum enzymatic activity of CAT and GPx was investigated by the colorimetric method (Biocore Diagnostik Ulm GmbH kits, Germany). The intra-assay coefficient of variation of CAT and GPx was 3.8% and 5.7% and the inter-assay coefficient of variation was 9.9% and 7.2%, respectively. Enzymatic activity of SOD was also determined by colorimetric method (BioVision kits, USA). The aforementioned measurements were carried out by an autoanalyzer device (BT-3000 (plus), Biotecnica, Italy). The intra-assay coefficient of variation was 3.2%, and the inter-assay coefficient of variation was 3.7%. FRAP was measured with spectrophotometry, as described by Benzie and Strain [41]. Based on this method, FRAP reagent was prepared with mixing 300 mmol/L of acetate buffer (pH: 3.6), 10 mmol/L of tripyridyl triazine (TPTZ) in 40 mmol/L HCL, and 20 mmol/L FeCl_3_·6H_2_O. Twenty-five *μ*L of serum was then added to 750 *μ*L FRAP reagent, and absorbance was recorded at 593 nm. Readings were expressed in *μ*mol/L. We drew the calibration curve of FRAP. Necessary dilution was done to ensure the FRAP value fell in the linear range of the standard curve. Mean and standard deviation were calculated. The intra-assay coefficient of variation was 3%, and the inter-assay coefficient of variation was 4.2%.

### 2.7. Statistical Analysis

Data were analyzed using SPSS software (version 16.0: SPSS Inc., Chicago, IL, USA). The Smirnov-Kolmogorov test was employed to test the normality of the variables in each group. The continuous variables were expressed as means ± standard deviation (SD), and categorical variables were presented as numbers and percentages. For comparison of baseline redox biomarkers of healthy controls with patients before and after surgery, an independent *t*-test was employed. A paired *t*-test was used to evaluate the mean difference between preoperation and postoperation measurements in patients. Cohen's *d* effect size was also employed to measure the magnitude of the differences. Evaluation of effect size is important because while the dependent *t*-test tells us whether differences between group means are real, it does not show the “size” of the difference [42, 43]. Descriptors for magnitudes of *d* = 0.01 to 2.0 are categorized into very small (*d* = 0.01), small (*d* = 0.2), medium (*d* = 0.5), large (*d* = 0.8), very large (*d* = 1.2), and huge (*d* = 2) [43, 44]. The Pearson correlation coefficient was deployed to examine the strength and direction of the interrelationships between continuous variables. The point-biserial correlation coefficient was used to assess the correlation between a continuous variable and a binary one. In all analyses, *p* value of less than 0.05 was considered statistically significant.

## 3. Results

**Figure 1:**
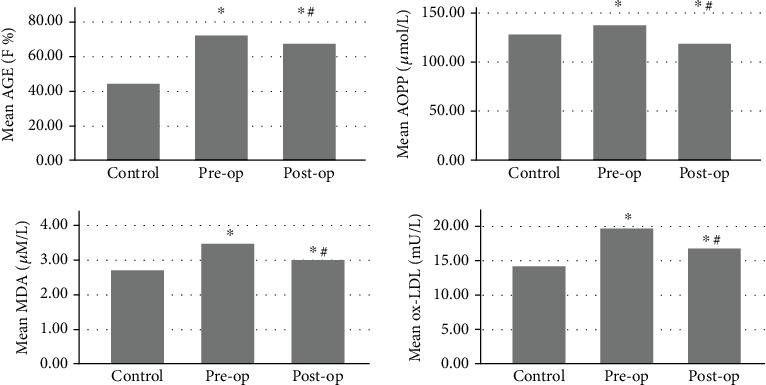
Comparison of oxidative damage markers in healthy controls and patients with colorectal cancer in pre- and postoperation conditions. ^∗^*p* < 0.05 vs. healthy control. ^#^*p* < 0.05 vs. pre-op condition.

**Figure 2:**
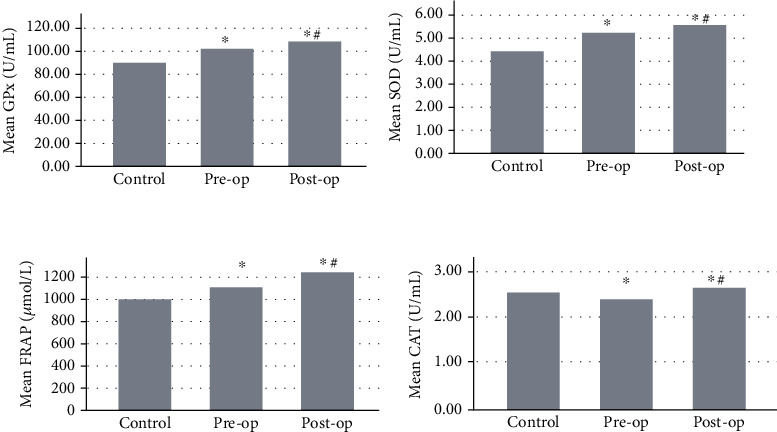
Comparison of antioxidant activity in healthy controls and patients with colorectal cancer in pre- and postoperation conditions. ^∗^*p* < 0.05 vs. healthy control. ^#^*p* < 0.05 vs. pre-op condition.

Baseline characteristics of the study population and tumor characteristics in patients are presented in Table 1. There were no significant differences between groups with respect to age, gender, BMI, and systolic and diastolic blood pressure. Comparison of lipid peroxidation products (ox-LDL and MDA), protein oxidation products (AOPP and AGEs), enzymatic antioxidants (SOD, GPx, and CAT), and antioxidant index (FRAP) in healthy controls and patients before and after surgery are displayed as bar charts in Figures 1 and 2.

### 3.1. Comparison of Control and Preoperative Conditions

Markers of oxidative damage including AGEs (72.49 ± 4.7 vs. 44.62 ± 7.08, *p* < 0.001), AOPP (137.64 ± 21.9 vs. 129.1 ± 12.1, *p* = 0.014), MDA (3.56 ± 0.30 vs. 2.74 ± 0.37, *p* < 0.001), and ox-LDL (19.78 ± 0.97 vs. 14.43 ± 1.17, *p* < 0.001) in patients before surgery were significantly higher than those in healthy controls. The antioxidant activity levels of GPx (102.37 ± 6.58 vs. 90.1 ± 7, *p* < 0.001) and SOD (5.13 ± 0.39 vs. 4.34 ± 0.35, *p* < 0.001) and antioxidant index of FRAP (1097.5 ± 156 vs. 1000 ± 213.9, *p* = 0.013) before surgery were significantly higher than control levels. CAT activity levels were significantly lower in preoperative condition than the control group (2.34 ± 0.34 vs. 2.48 ± 0.36, *p* < 0.001) (Table 2).

### 3.2. Comparison of Preoperative and Postoperative Conditions

There were significant differences between baseline measurements of oxidative stress markers before surgery and their values after surgery (Table 3). AGE (72.49 ± 4.7 vs. 67.93 ± 8.8, *p* < 0.001), AOPP (137.64 ± 21.9 vs. 119.08 ± 33.1, *p* < 0.001), MDA (3.56 ± 0.30 vs. 3.05 ± 0.33, *p* < 0.001), and ox-LDL (19.78 ± 0.97 vs. 16.94 ± 1.02, *p* < 0.001) concentrations reduced significantly after operation. The largest effect sizes were seen in lipid peroxidation products including ox-LDL (effect size = −2.853, 95% CI 2.50-3.19) and MDA (effect size = −1.617, 95% CI 0.43-0.57). On the other hand, a significant rise was seen after surgery in FRAP (1097.5 ± 156.7 vs. 1239.3 ± 290, *p* < 0.001) and activity of enzymatic antioxidants including CAT (2.34 ± 0.34 vs. 2.63 ± 0.38, *p* < 0.001), GPx (102.37 ± 6.58 vs. 108.03 ± 6.95, *p* < 0.001), and SOD (5.13 ± 0.39 vs. 5.53 ± 0.31, *p* < 0.001). The largest effect sizes among antioxidants were seen in SOD (effect size = 1.135, 95% CI 0.46-0.34) and GPx (effect size = 0.836, 95% CI 8.65-2.67) (Table 3).

### 3.3. Comparison of Control and Postoperative Conditions

Despite the reduced amounts of oxidative damage markers after surgery, they were still higher than normal levels (AGEs (67.93 ± 8.8 vs. 44.62 ± 7.08, *p* < 0.001), MDA (3.05 ± 0.33 vs. 2.74 ± 0.37, *p* < 0.001), and ox-LDL (16.94 ± 1.02 vs. 14.43 ± 1.17, *p* < 0.001)). Postoperative AOPP levels were less than control amounts (119.08 ± 33.1 vs. 129.1 ± 12.1, *p* = 0.035). Activity of GPx (108.03 ± 6.95 vs. 90.1 ± 7, *p* < 0.001), SOD (5.53 ± 0.31 vs. 4.34 ± 0.35, *p* < 0.001), and CAT (2.63 ± 0.38 vs. 2.48 ± 0.36, *p* = 0.045) and antioxidant index of FRAP (1239.3 ± 290 vs. 1000 ± 213.9, *p* < 0.001) after surgery remained significantly higher than control levels (Table 2).

### 3.4. Redox Biomarker and Clinical Parameters

Regarding the association of redox biomarkers with tumor size and stage in patients with CRC before surgery, we found no statistically significant correlation between each redox biomarker with tumor size and cancer stage. We displayed all correlation related data in Table 4.

## 4. Discussion

### 4.1. Redox State in Cancer

**Figure 3:**
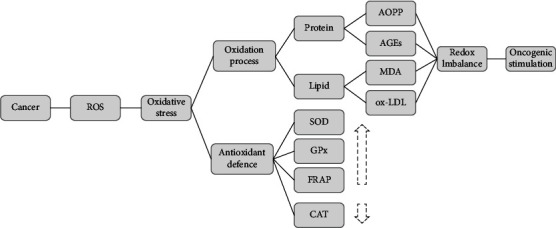
The effect of cancer-induced oxidative stress on redox homeostasis.

Our findings demonstrated that patients with colorectal cancer have higher levels of oxidative damage biomarkers and antioxidant activity, except for CAT, compared to healthy controls (Figure 3). Growing evidence suggests that cancer cells in general are under increased oxidative stress compared to normal cells [45]. The redox status in CRC is tightly associated with the tumor environmental trait, inflammatory infiltration, and tumor budding which is defined as a detached cluster of fewer than 5 cells at the invasive front of a tumor [46, 47]. It has been indicated that CRC with high grade of budding generally presented with higher levels of oxidative stress [46]. While increased oxidative stress drives the activation of death pathways in normal cells, conversely, malignant cells exploit oxidative background for their advantage [15]. Antioxidant defense and repair activities rise in redox imbalance, but not enough to cope with reactive species in cancer milieu and could be quickly inactivated or impaired by existent oxidative stress [48, 49]. ROS-induced oxidative damage of DNA, proteins, and lipids in cancer cells facilitates escape from apoptosis and produces a proliferation potential through activation of cell survival signals [50]. Uncontrolled cell growth and proliferation in cancer cells require high levels of ATP supply. This energy demand in general, places a further stress on the mitochondrial respiration chain and consequently leads to increased ROS generation [45]. Mitochondria as the powerhouse of the cell are extremely susceptible to oxidative stress [51, 52]. ROS stimulates lipid peroxidation in mitochondria and results in suppression of mitochondrial metabolism and intracellular redox disequilibrium [53]. Lipid peroxidation products including MDA and ox-LDL can seriously damage DNA and inhibit DNA repair capacity through their direct interaction with repair proteins [54]. ox-LDL is a potent and independent mitogenic factor and could contribute to the progression of carcinoma by increasing the expression of cell cycle-activating proteins and release of cytokines and growth factors [54–58]. Multiple studies reported elevated levels of serum ox-LDL levels in colon, breast, and ovarian cancers when compared to controls [54, 56, 59]. Suzuki et al. study showed that the higher levels of serum ox-LDL may increase risk of colorectal cancer [60]. However, obtained results from Diakowska et al.'s research demonstrated that serum ox-LDL and antibodies against ox-LDL were not satisfactory risk markers of CRC [61]. MDA as the most mutagenic product of lipid peroxidation participates in cell proliferation and differentiation, as well as in apoptosis pathways [62]. Increased plasma or urine MDA concentration has been observed in many cancers including breast and colorectal cancer in previous studies [63–67]. In contrast, Chang et al.'s study showed lower levels of MDA in CRC [68]. To fulfill high ATP demands, cancer cells uptake glucose at a higher rate and enhance glycolysis. However, insufficient oxygen supplies lead to a shift toward anaerobic metabolism and consequently AGE formation [69, 70]. Increased AGE production results in tumor initiation, growth, and invasion through increased cell proliferation and migration [71, 72]. Therapeutic suppression of AGEs could have potential for clinical benefit in cancer [73]. Previously, Heijst et al.'s study showed that one of the highest expressions of AGEs was found in adenocarcinoma of the colon compared to other cancer cells [69]. AOPP as a reliable marker of protein oxidation mostly represents aggregates of albumin damaged by oxidative stress [74]. Albumin degradation secondary to protein oxidation plays an important role in the hypoalbuminemia and increased AOPP levels of colorectal cancer patients [75]. Previous studies showed higher levels of AOPP in patients with cancers compared to healthy controls [54, 66, 68, 76]. Veljković et al.'s study showed that the level of AOPP in the tumor tissue was significantly higher in comparison to the healthy colon tissue [77].

Protection of cell constituents from oxidative stress can be accomplished through enzymatic and nonenzymatic mechanisms. Nonenzymatic antioxidants such as FRAP can be described as reductants, and inactivation of oxidants by reductants can be described as redox reactions [41]. FRAP is a measure of the antioxidant power, based on the reduction of ferrous ions by the effect of the reducing power of plasma constituents [50]. Enzymatic defense mainly consists of SOD, GPx, and catalase. SOD catalyzes the dismutation of superoxide anions to hydrogen peroxide, which is metabolized by catalase and GPx [78]. Multiple studies reported a considerable increase in SOD and GPx activity of specimens obtained from tumors when compared to nontumorous colorectal tissues [51, 64, 77, 79, 80]. GPx2, a gastrointestinal tract GPx isoform, overexpression was observed in gastric cancer (both primary and metastatic foci) [81] and colorectal cancer tissue [82] when compared with normal tissue. Catalase is localized mainly in peroxisomes and decomposes H_2_O_2_ to water, acting in concert with SOD [78, 83]. Previous studies showed that the catalase activity and peroxisomes both are significantly reduced in tumors of colon, liver, and lung [66, 83–85]. Glorieux and collaborators have suggested that, in cancer cells exposed to oxidative stress, phosphorylation of catalase would occur and result in decreased catalase activity [86]. In parallel with our results, Skrzydlewska et al.'s study reported a significant increase in the activity of Cu, Zn-SOD, and GPx as well as a decrease in CAT activity in all clinical stages of colorectal cancer patients as compared to the control group [64]. Zińczuk et al.'s study found lower activity of CAT, GPx, and FRAP alongside with higher activity of SOD and higher concentrations of AGEs, AOPP, and MDA in CRC patients compared to the healthy control [66]. Chang et al.'s study showed that the activities of SOD, GPx, and CAT were decreased significantly in CRC patients [68].

This study did not observe any significant association between redox biomarkers and the size and stage of the tumor. These results may underline the point that cancer cells are not the only source of oxidative stress. Within TME, oxidative stress can have intrinsic or extrinsic origin. Oxidative stress generation is the result of dynamic interactions of cancer cells with their microenvironment. Several stromal factors such as cancer-associated fibroblast and macrophage or hypoxia have already been proven to directly produce ROS and elicit a prooxidant atmosphere. Cancer cells exacerbate oxidant environment by intrinsic production of oxidative stress [15]. In line, Satomi et al.'s study found no significant correlation of colorectal tumor size (maximum diameter) with SOD activity and no significant difference in SOD activity according to the stage of disease [80].

### 4.2. Redox State after Cancer Surgery

After surgery, we observed significant drops in levels of oxidative damage biomarkers; however, they were still higher than healthy control results. At the same time, there was a substantial rise in the activity of antioxidant biomarkers (Figures 1 and 2). Decreased levels of oxidative damage biomarkers as well as increased activity of antioxidants after tumor resection surgery indicated that resected tumor most probably was the major source of oxidative stress and the main culprit in disruption of antioxidant defense. However, systemic oxidative stress was still higher than healthy controls after tumor resection and this probably addresses the underlying increased oxidative stress [87]. Surgery stress as a confounder may obscure the real effect of tumor cytoreductive procedure on redox outcome. Hence, further improvement in redox homeostasis could be expected with dwindling of surgery-induced oxidative stress over time. Hristozov et al.'s study explored the oxidative stress in erythrocytes of patients with different cancers before and after surgery, and they found no significant difference in SOD, significant rise in CAT, and remarkable drop in MDA [88]. Ahmad et al.'s findings showed higher levels of SOD, CAT, and glutathione-S-transferase (GST) and lower levels of MDA after benign prostatic hyperplasia surgery compared to presurgery condition [89].

At high levels, ROS promote severe cellular damage. Cancer cells need to combat high levels of oxidative stress by evolving powerful antioxidant system to regulate ROS to levels that are compatible with their cellular biological functions [14]. The enhancement of the antioxidant enzyme activity is a result of the activation of genes that code for antioxidant enzymes by oxidants and is an outcome of cellular adaptation to conditions of increased oxidative stress [79]. Targeting enhanced antioxidant defense which could be considered as an Achilles heel for tumor cells may represent a strategy that can specifically kill cancer cells while sparing normal cells and has had promising results in in vitro and in vivo studies [14, 90–92]. On the other hand, the antioxidant defense systems in noncancerous tissue are vulnerable to damage by active oxygen [80]. Increased and long-term oxidative stress results in the suppression of antioxidant defense by exhaustion of enzymes owing to ROS scavenging or the inhibition of enzymes caused by ROS in normal cells [93]. What we are witnessing after surgery are noncancerous cells that employ their recovered antioxidant defense to cope with underlying stress including surgery. After tumor resection, the noncancerous cells in an environment away from continuous ROS bombardment exhibit higher levels of antioxidant activity compared to presurgery condition. Altogether, our findings suggest that removal of TME as the major source of oxidative stress by cytoreductive surgery could potentially improve redox homeostasis and enhance antioxidant defense.

Cytoreductive surgery is an approach to cancer treatment that is aimed at reducing the number of cancer cells via resection of primary tumor or metastatic deposits [94]. This procedure is a part of treatment modalities of colorectal cancer nowadays that make adjuvant therapy more productive and improve the length of survival [95]. The TME is the main obstacle for the efficacy of adjuvant therapy, as impaired blood flow of growing tumor leads to hypoxia, acidity, and reduced accessibility of any drug [96, 97]. Overcoming microenvironment-related resistance may have a fundamental impact on treatment efficacy and patient outcome [97]. Accordingly, while recurrent and metastatic colon cancers have routinely not been treated with surgery, there is an increasing body of literature supporting the role of surgical intervention for metastatic colon cancer [98].

### 4.3. Effect Size of Cancer Surgery on Redox

The largest effect sizes we obtained for cytoreduction-induced redox alterations were in lipid peroxidation products (ox-LDL and MDA with huge and very large negative effect sizes, respectively) and in antioxidant activity of SOD and GPx (both with large positive effect sizes). To the best of our knowledge, this is the first study estimating the effect size of tumor resection surgery on improvement of redox homeostasis in patients with colorectal cancer. The results may suggest the probable ameliorating effect of cytoreductive surgery on dysfunctional mitochondria as a source and target of lipid peroxidation products [99] in colorectal cancer. Since our study focused on evaluation of redox biomarkers in general and has not included mitochondrial redox measurements, the results may provide only some clues that could serve as a basis for future mitochondrial research. Targeting different aspects of mitochondrial metabolism that contribute to redox regulation has been proven to be a successful anticancer strategy [90]. Sánchez-Aragó et al.'s study showed that colon cancer progression is only possible when cancer cells repress the biogenesis and functional activity of mitochondria [100].

### 4.4. Limitations

The principal limitation of the present study is the strict inclusion criteria that led to difficulties in enrolling patients and reduced the generalizability of the study findings to the target population. However, the stringent inclusion criteria improve study population homogeneity and statistical power and could be the strength of our study at the same time [101]. Another limitation is that we narrowly evaluated redox status early (24 h) after tumor resection surgery. Future studies with long-term prospective follow-up would be needed to assess the time course of oxidative stress after cytoreductive surgery.

## 5. Conclusion

In summary, this study indicated that patients with colorectal cancer had higher levels of oxidative stress and antioxidant activity compared to healthy controls. After surgical resection, we observed a significant decline in oxidative stress biomarkers with the largest effect size in lipid peroxidation products alongside with a significant rise in antioxidant activity. Hence, reducing the fire flame of oxidative stress through cytoreductive surgery possibly enhances antioxidant defense and subsequently may attenuate tumor progression in colorectal cancer. Further studies taking into account all factors involved in systemic oxidative stress are warranted to elucidate the value of cytoreductive surgery in the improvement of redox homeostasis, response to adjuvant therapy, and long-term prognosis in patients with colorectal cancer.

## Figures and Tables

**Table 1:** Basic characteristics of patient and control groups and tumor characteristics in patients.

	Patients (*n* = 60)	Controls (*n* = 43)	*p* value
Gender (F/M) (*N*)	30/30	23/20	NS
Age (yr.)	50.28 ± 8.2	52.73 ± 1.72	NS
Weight (kg)	79.70 ± 13.9	79.68 ± 6.63	NS
BMI (kg/m^2^)	31.24 ± 5.6	29.8 ± 5.1	NS
SBP (mmHg)	120.5 ± 11.1	120 ± 11.5	NS
DBP (mmHg)	78.58 ± 4.8	79.68 ± 6.63	NS
Tumor characteristics			
Stage of dx, *n* (%)			
І	30 (50)		
II	30 (50)		
Lymph nodes			
Negative	60 (110)		
Positive	0 (0)		
Histological type, *n* (%)			
Adenocarcinoma	53 (88.3)		
Mucinous adenocarcinoma	7 (11.7)		
Pathological grading, *n* (%)			
Well/moderate	60 (100)		
Poor/undifferentiated	0 (0)		
Tumor size (cm)	4.09 ± 0.47		
Location of tumor, *n* (%)			
Colon	23 (38.3)		
Rectum	16 (26.6)		
Sigmoid	14 (23.3)		
Cecum	7 (11.6)		
Mode of detection			
Screening	5 (8.3)		
Clinical	55 (91.7)		

Data is presented as mean ± SD or number and percent. BMI: body mass index; SBP: systolic blood pressure; DBP: diastolic blood pressure; NS: nonsignificant.

**Table 2:** Comparison of redox biomarkers in healthy controls and patients with colorectal cancer before and after surgery.

Markers	Healthy control	Before surgery	*p* value	Healthy control	After surgery	*p* value
AGE (F %)	44.62 ± 7.08	72.49 ± 4.7	<0.001	44.62 ± 7.08	67.93 ± 8.8	<0.001
AOPP (*μ*mol/L)	129.1 ± 12.1	137.64 ± 21.9	0.014	129.1 ± 12.1	119.08 ± 33.1	0.035
MDA (*μ*M/L)	2.74 ± 0.37	3.56 ± 0.30	<0.001	2.74 ± 0.37	3.05 ± 0.33	<0.001
ox-LDL (mU/L)	14.43 ± 1.17	19.78 ± 0.97	<0.001	14.43 ± 1.17	16.94 ± 1.02	<0.001
FRAP (*μ*mol/L)	1000 ± 213.9	1097.5 ± 156	0.013	1000 ± 213.9	1239.3 ± 290	<0.001
CAT (U/mL)	2.48 ± 0.36	2.34 ± 0.34	0.049	2.48 ± 0.36	2.63 ± 0.38	0.045
GPx (U/mL)	90.1 ± 7	102.37 ± 6.58	<0.001	90.1 ± 7	108.03 ± 6.95	<0.001
SOD (U/mL)	4.34 ± 0.35	5.13 ± 0.39	<0.001	4.34 ± 0.35	5.53 ± 0.31	<0.001

Data is presented as mean ± SD. AGE: advanced glycation end product; AOPP: advanced oxidation protein products; CAT: catalase; FRAP: ferric reducing ability of plasma; GPx: glutathione peroxidase; MDA: malondialdehyde; ox-LDL: oxidized low density lipoprotein; SOD: superoxide dismutase.

**Table 3:** Alterations in redox biomarkers after surgery in patients with colorectal cancer.

Markers	Before surgery	After surgery	Mean difference	95% CI	Cohen's *d*	*p* value
AGE (F %)	72.49 ± 4.7	67.93 ± 8.8	4.56	2.34-6.77	-0.646	<0.001
AOPP (*μ*mol/L)	137.64 ± 21.9	119.08 ± 33.1	18.56	10.09-27.03	-0.661	<0.001
MDA (*μ*M/L)	3.56 ± 0.30	3.05 ± 0.33	0.50	0.43-0.57	-1.617	<0.001
ox-LDL (mU/L)	19.78 ± 0.97	16.94 ± 1.02	2.84	2.50-3.19	-2.853	<0.001
FRAP (*μ*mol/L)	1097 ± 156.7	1239.3 ± 290	-141.71	-(218-65.1)	0.608	<0.001
CAT (U/mL)	2.34 ± 0.34	2.63 ± 0.38	-0.29	-(0.35-0.23)	0.804	<0.001
GPx (U/mL)	102.37 ± 6.58	108.03 ± 6.95	-5.66	-(8.65-2.67)	0.836	<0.001
SOD (U/mL)	5.13 ± 0.39	5.53 ± 0.31	-0.40	-(0.46-0.34)	1.135	<0.001

Data is presented as mean ± SD. AGE: advanced glycation end product; AOPP: advanced oxidation protein products; CAT: catalase; FRAP: ferric reducing ability of plasma; GPx: glutathione peroxidase; MDA: malondialdehyde; ox-LDL: oxidized low density lipoprotein; SOD: superoxide dismutase.

**Table 4:** Correlation coefficients between redox biomarkers and tumor size and point-biserial correlation between cancer stages (as stage І and II) and redox biomarkers in patients with colorectal cancer.

Redox biomarkers	Tumor size (cm)		Cancer stage (І & II)	
	*r*	*p* value	*r*	*p* value
AGE (F %)	0.111	NS	0.015	NS
AOPP (*μ*mol/L)	0.219	NS	-0.172	NS
MDA (*μ*M/L)	0.177	NS	0.245	NS
ox-LDL (mU/L)	0.013	NS	0.133	NS
FRAP (*μ*mol/L)	0.021	NS	0.087	NS
CAT (U/mL)	0.011	NS	-0.164	NS
GPx (U/mL)	0.069	NS	0.196	NS
SOD (U/mL)	0.201	NS	0.064	NS

AGE: advanced glycation end products; AOPP: advanced oxidation protein products; CAT: catalase; FRAP: ferric reducing ability of plasma; GPx: glutathione peroxidase; MDA: malondialdehyde; ox-LDL: oxidized low-density lipoprotein; SOD: superoxide dismutase; NS: nonsignificant.

## Data Availability

The original data can be requested from the corresponding author.
